# Use of general practitioner services among high-cost older patients in somatic hospitals – a Norwegian registry-based study

**DOI:** 10.1186/s12913-026-14924-1

**Published:** 2026-06-13

**Authors:** Morten Lønhaug-Næss, Monika Dybdahl Jakobsen, Bodil H. Blix, Karl Ove Hufthammer, Jill-Marit Moholt

**Affiliations:** 1 Department of Health and Care Sciences, Faculty of Health Sciences, UiT The Arctic University of Norway, Tromsø, Norway; 2 Centre for Care Research North, Department of Health and Care Sciences, Faculty of Health Sciences, UiT The Arctic University of Norway, Tromsø, Norway; 3 Centre for Care Research East, Department of Health Sciences in Gjøvik, Norwegian University of Science and Technology (NTNU), Gjøvik, Norway; 4 Centre for Care Research West, Western Norway University of Applied Sciences, Bergen, Norway; 5 Division of Rehabilitation Services, University Hospital of North Norway, Tromsø, Norway

**Keywords:** Older adults, High-cost older patients, General practitioner, Hospitals, Municipal healthcare, Healthcare service utilization, Universal healthcare, Norway

## Abstract

**Background:**

General practitioners (GP) play a vital role in ensuring comprehensive and integrated healthcare to older adults with substantial use of somatic hospitals services. These patients, often referred to as high-cost older patients, use a substantial proportion of specialized healthcare resources, but less is known about their use of primary care and GP services. This study aims to explore the frequency of the use of GP services among high-cost older patients and other older patients admitted to Norwegian somatic hospitals and identify characteristics associated with the frequency of service use.

**Methods:**

This is an observational cross-sectional study using registry data of older Norwegian patients (≥65 years) with ≥1 unplanned contact with somatic hospitals in 2019 (*n* = 212,022). We used a negative binomial regression model to analyze the frequency of use of GP services and associations with patient characteristics. GP services include all contact types (consultations, home visits, interprofessional contacts, brief contacts, and administrative contacts). Both adjusted and unadjusted results were reported to provide a comprehensive understanding of the data. The high-cost group was defined as patients with the 10% highest diagnosis-related-group weights.

**Results:**

A total of 183,847 patients, consisting of 18,440 high-cost and 165,407 non-high-cost older patients were included in the regression analysis. The unadjusted average frequency of GP contacts was 24.4 for the high-cost group and 16.6 for the non-high-cost group. After adjusting for the different characteristics in the two groups, the high-cost group still had a higher frequency of GP contacts compared to the non-high-cost older group. Several characteristics were associated with higher expected frequency of GP contacts, including older age, being female, and having multiple main diagnoses, multiple contacts with out-of-hours services, and/or longer hospital stay.

**Conclusion:**

The high-cost group had higher utilization of GP services across various contact types compared to other older adults. The healthcare utilization of high-cost older patients appears to transcend the context of somatic hospitals, as they could be deemed frequent users of GP services. Additionally, the association between GP service use and patient characteristics might indicate that groups known to have higher healthcare needs also use more services.

## Background

General practice is an important part of most primary health services and refers to medical services provided by physicians who specialize in general practice or family medicine. In addition to managing acute medical needs, general practitioners (GPs) provide a broad range of essential services, including preventive care, such as routine check-ups, vaccinations and health screenings to detect health issues early. GPs also play a crucial role in coordinating care, ensuring consistent follow-ups and treatments, which helps prevent complications in chronic disease management [1–3]. Additionally, as gatekeepers to specialized services, GPs evaluate the need for hospital care, ensuring referrals are made when necessary [4]. Previous research has shown that regular use of GP services can reduce the frequency and costs of hospital admissions. According to Blinkenberg et al. (2019), timely intervention by GPs can prevent emergency hospital admissions. Acting as gatekeepers, GPs often address health issues at an earlier stage, mitigating the need for acute hospital care, which is typically more resource intensive and costly [5]. A study by Skarshaug et al. [6] found that when patients experienced GP continuity, healthcare utilization, particularly hospital admissions, was lower. Lastly, Hansen et al. [7] also demonstrated that gatekeeping and continuity in GP care led to reduced specialized healthcare use, contributing to lower costs and less frequent hospitalizations. In contrast, research has indicated that patients often rely on frequent contacts with multiple healthcare providers in healthcare systems where GPs do not have a clear gatekeeping role, which may potentially lead to poorer health outcomes such as delayed diagnoses, fragmented care and higher incidences of medication error [8].

The gatekeeping role of GPs in Norway introduces complex dynamics that can lead to both increased and decreased hospitalizations. Although this role is generally intended to reduce hospital admissions, this may not necessarily be the case for patients with complex health problems, which often include multiple chronic illnesses, frailty, and age-related health issues, often seen among older adults [9, 10]. Condelius et al. [11] emphasize that hospital admission among older adults is closely linked to the effectiveness of coordination between primary and specialized healthcare sectors, which can vary greatly and impact patient outcomes.

High-cost older patients constitute a small group of older patients admitted to Norwegian somatic hospitals. They often have complex medical conditions that lead to significant utilization of hospital economic resources [12]. As a result, high-cost populations have gained attention from healthcare authorities, policy makers and researchers in recent years. Efforts are increasingly focused on understanding and addressing the needs of this population to find strategies to optimize care and improve health outcomes. To our knowledge, there are currently no studies in Norway on the use of GP services among high-cost older patients in somatic hospitals. As primary healthcare services, including GP services, play a vital role in ensuring comprehensive and integrated healthcare, it is of interest to examine the use of healthcare services outside of somatic hospitals for these patients. This insight may add to the existing knowledge that can be used to identify opportunities for optimizing care for these patients across healthcare levels. Additionally, while international studies have examined the association between sociodemographic status and the use of hospital services for high-cost older patients [13, 14], few studies have investigated the association between these characteristics and high-cost older patients’ use of GP services [9, 15]. Based on these considerations, this study aims to explore the frequency of the use of GP services among high-cost older patients and other older patients admitted to Norwegian somatic hospitals. We also aim to identify characteristics associated with the frequency of service use.

## Methods

This is a population-based cross-sectional study in which we use data from three Norwegian registries. The Norwegian Patient Registry (NPR) provides information on sex, age, and diagnosis-related group (DRG) weight, number of main diagnosis groups, and length of stay (LOS) in somatic hospitals [16]. Statistics Norway (SSB) collects sociodemographic information on education level, household income, marital status, and the index of municipal centrality. The Norwegian Registry for Primary Health Care (NRPHC) contains information about everyone who receives health and care services from the municipal authority [17]. It includes data from the Norwegian Control and Payment of Health Reimbursements Database (KUHR), which contains information about the service utilization among municipal emergency medical centers and GPs [18].

### Setting: General practice in Norway

The organization of GP services differs across healthcare systems, and countries with universal healthcare often adopt a community-based approach to ensure easy access to primary care [19]. In Norway, all residents registered in the National Population Registry are entitled to GP services, which are provided at the municipal level as part of the primary healthcare sector. Residents have a right to a GP, and municipalities are legally obligated to offer necessary GP services [20]. This structure aligns with Norway’s principles of universal, equitable, and accessible healthcare [1, 21, 22]. Norwegian GPs operate under a mixed payment system that combines a per capita component and a fee-for-service model. For each registered patient, GPs receive a fixed per capita payment from the government, which supports the provision of ongoing general care. In addition, GPs are reimbursed on a fee-for-service basis, which consists of state reimbursements for specific medical services rendered, such as consultations or procedures. They also receive a small copayment directly from the patient for each visit, adding a direct fee-for service component [1]. This mixed system aims to balance the incentive for GPs to manage a stable patient list while providing appropriate compensation for specific services delivered [23]. In 2019, 98.6% of all Norwegians had access to a GP, and only 9 out of 356 municipalities lacked GP services . Older adults aged 65–79 years had approximately 4 GP consultations on average, while older adults aged 80–89 years and 90 years and above had an average of 5 and 3 consultations, respectively [24]. GPs in rotation also serve municipal emergency medical centers to provide out-of-hours GP services [4]. These centers offer immediate medical assistance for non-life-threatening emergencies, illnesses, and injuries outside of the GP’s regular office [1], and may refer patients to hospital if needed.

### Study population: categorization of high-cost and non-high-cost older patients

Norwegian hospitals use DRG as a system for classifying patients based on diagnoses, procedures, age, sex, and discharge status. DRG provides a standardized way to determine hospital reimbursements from the state for different patient groups. It serves as a framework for understanding resource utilization within Norwegian somatic hospitals by categorizing the complexity of hospital stays. DRG weights are relative measures, reflecting the resource use associated with a particular group of patients compared to the average patient [25], but do not directly measure patients’ underlying health status or healthcare needs. Rather, they quantify the hospital resources utilized during episodes of care. In this study, we define the high-cost older patient group as the 10% of participants with the highest cumulative DRG weights over the course of a year, and the non-high-cost older patient group as the remaining 90% of the other adult older patients. The decision to use the top 10% as a threshold is methodological, guided by a previous study [26], and facilitates a clear comparison between the highest-cost patients and the rest of the study population. This 10% threshold is commonly used in research on healthcare costs [9, 27–29].

### Inclusion and exclusion criteria

**Fig. 1:**
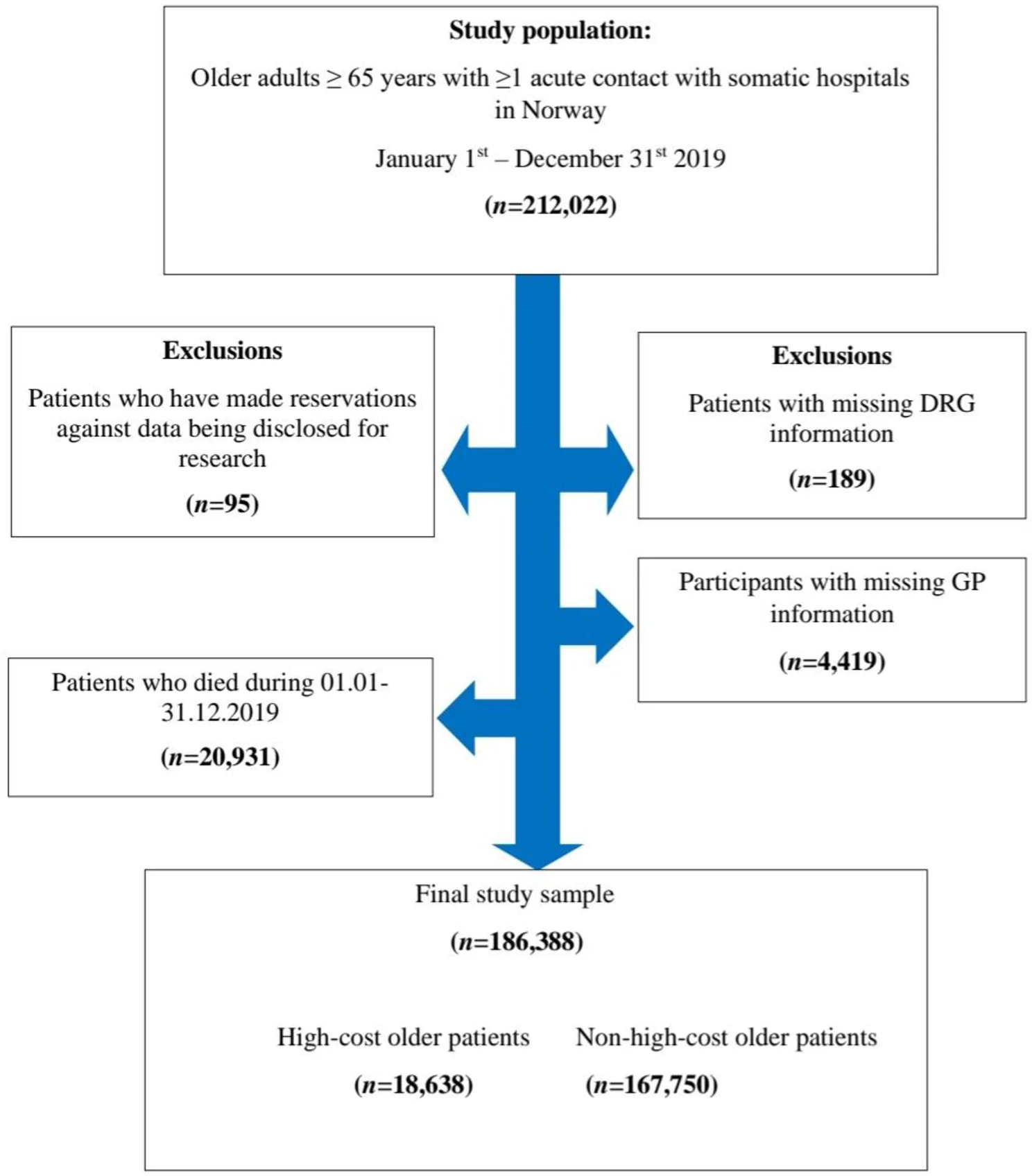
Flow chart for selection of the study sample, including exclusion criteria. In this figure show the study population and exclusion steps for the study sample

The study is part of a broader research initiative examining the municipal healthcare utilization among older adults with unplanned admissions to Norwegian somatic hospitals. The data contains information at an individual level for the year 2019, capturing all recorded contacts with somatic hospitals, GP services, and municipal emergency medical centers from 2019-01–01 to 2019–12-31. We included all older adults ≥ 65 years who were registered with at least one unplanned contact with somatic hospitals in Norway (*n* = 212,022). Unplanned contacts include day treatment, outpatient care and admittance to acute care wards. Patients who had an acute incident during a planned admission were also included in the study sample. The study sample accounts for 23% of total older adult population (aged 65 years and older) in 2019 [30]. Patients who had opted out of their information being used for research (*n* = 95) and patients with missing DRG information (*n* = 189) were excluded. We excluded patients with missing GP contact information (*n* = 4,419). Among these, 1,506 patients died during 2019 before visiting a GP. The remaining 2,913 patients had no recorded data on GP service utilization in the KUHR registry. The absence of GP data for these patients does not necessarily indicate a lack of GP visits but rather that their data were not available or captured in the registries. Furthermore, we excluded the remaining patients who died during 2019 (*n* = 20,931) as SSB does not collect income information for the year a person dies. The final study population consisted of 186,388 patients divided into high-cost (*n* = 18,638) or non-high-cost groups (*n* = 167,750) based on DRG weights. Figure 1 shows a flowchart for the study sample, including exclusion criteria.

### Utilization of GP services

GP service use is defined as the total number of contacts each participant had with a GP during 2019. There were also separate service use variables for different types of contact. NRPHC derived the variables containing GP contact information from tariffs/rates sent to the Norwegian Health Economics Administration (HELFO) from the GPs. The tariffs/rates are categorized into contact types such as consultations (scheduled or emergency), brief contacts (such as prescription renewal, sick leave without consultation, and test results), home visits, interprofessional contacts between the GP and other health professions, and administrative contacts/other contacts. The included GP contact types and the tariffs/rates associated with the codes are described in Appendix 1 [31, 32]. In the *main* regression analysis, we used the *total* number of GP contacts as the outcome, as this allows for a comprehensive picture of the participants’ healthcare utilization. As supplemental analyses, we conducted regression analyses for the various GP contact types.

### Sociodemographic and clinical characteristics

The included sociodemographic characteristics consisted of sex, age, educational level, household income, and the index of municipal centrality. These variables were selected as sociodemographic factors based on their relevance in previous studies and their potential influence on the use of GP services [33–35]. Sex was recorded as either male or female. Age groups were recorded in 5-year intervals ranging from 65 years to ≥90 years. Marital status was recorded as being married/cohabitant/partner or single, separated, divorced, or widowed, and the variable was recoded to a binary variable by combining the last four categories. Educational level was recorded using five categories as primary school, high school, vocational school, higher education up to three years, and higher education beyond four years. The variable was recoded into three categories, reflecting whether the individual had primary school, high school/vocational school, or higher education (college/university) attainment. Household income is the sum of all individual incomes of the household members and was recorded using six categories and recoded into three categories, low (≤450,000 NOK), moderate (451,000–750,000 NOK) or high (>750,000 NOK) income. The variable represents the sum of all individual incomes within the household after tax and does not specify whether income equivalents have been applied. Therefore, we assume that no adjustment for income equivalence has been made in this variable.

The index of municipal centrality compares the centrality of entire municipalities relative to each other. The variable was recorded using six categories ranging from the least to most central municipalities [36] and recoded into three broader categories: least central, mid-central, or most central. The least central municipalities are characterized by low proportion of urban population and long distances to workplaces, services and urban centers. Mid-central municipalities have a mixed urban and rural population, and moderate distances to urban centers, workplaces and service offerings. Most central municipalities are closest to urban centers and have the shortest distances to workplaces and services as well as the highest proportion of residents living in urban areas [36].

Clinical characteristics included the number of different main diagnosis groups, the number of contacts with municipal emergency medical centers, and the length of stay (LOS) in somatic hospitals. Guided by previous studies, the number of different main diagnosis groups was included as a proxy measure for multimorbidity [37, 38], reflecting whether the patient had several diagnoses across the various organ systems. Based on the 22 available main diagnosis groups from NPR (Appendix 2), we constructed a variable reflecting whether patients had 1, 2, 3, 4 or ≥ 5 different main diagnoses registered in 2019. We included the total number of contacts with municipal emergency medical centers in 2019 to adjust for patients who might have had underlying health conditions predisposing them to out-of-hours contact with a GP. The variable was recoded into categories (no contacts, 1, 2, 3, 4, or ≥ 5 contacts). We also included LOS to control for the potential impact of hospitalizations on the use of GP services. The variable was recoded into the categories ‘<1 week (0–6 days)’, ‘1–2 weeks (7–13 days)’, ‘2–3 weeks (14–20 days)’, ‘3–4 weeks (21–27 days)’, and ‘≥4 weeks (≥28 days)’.

### Statistical analysis

We used descriptive statistics and bivariate analyses to provide an overview of the study sample. Separate analyses were conducted for the high-cost and non-high-cost groups. The Mann–Whitney U test was used to compare the distribution of GP contacts between the high-cost and non-high-cost groups across different characteristics. We used a negative binomial regression model to investigate the associations between the number of GP contacts and sociodemographic and clinical factors. This model was chosen due to overdispersion of the data, making a simple Poisson model unsuitable. Furthermore, we performed supplemental analyses for the subgroups of GP contacts, focusing on different categories of GP contacts, including brief contacts, consultations, and interprofessional contacts. For all regression analyses, we report the exponentiated coefficients, exp(*β*), which are estimated incidence rate ratios. For example, if the exp(*β*) for one level of an explanatory variable is 1.30, a patient with *this level* of the variable is expected to have 30% more contacts in one year than a patient with the same characteristics but having the *reference level* of the given explanatory variable. The significance level was set to 0.05, and all reported confidence intervals (CIs) are 95% CIs. The statistical analyses were conducted using SPSS version 29.0 and R version 4.3.2.

### Missing data

In the final study sample, 2,541 (1.4%) patients had missing data on either household income, educational level, marital status, or index of municipality centrality (198 high-cost older patients and 2,343 non-high-cost older patients). Due to the very low level of missing data, we used complete-case analyses for all regression models.

### Sensitivity analysis

As a sensitivity analysis, we fitted the main regression model but excluded outliers – participants with substantially more GP contacts (≥50 contacts) than the rest of the study population. This was done because we suspected that the model might not fit well for these participants and that their high number of contacts might have an unduly large effect on the coefficient estimates. We also conducted a sensitivity analysis to examine the potential impact on the regression model of including participants who had died. The model was run without the variable for income information as SSB only collects household income data for living participants registered with an address at the end of the year.

## Results

Descriptive statistics for GP use for the high- and non-high-cost groups are presented in Table 1. The analysis of the total number of GP contacts indicates that the high-cost group had on average approximately 8 more contacts in 2019 than the non-high-cost group. In addition, the high-cost group used on average more GP services across all contact types than the non-high-cost group. The most frequently used GP services among both groups were brief contacts, consultations, and interprofessional contacts.

**Table 1:** Descriptive statistics for all contact types with a GP (*n* = 186,388)

	High-cost older patients (n = 18,638)		Non-high-cost older patients (n = 167,750)	
	Mean (SD)	95% CI	Mean (SD)	95% CI
Total number of GP contacts^**^	24.4 (18.6)	24.1–24.6	16.6 (14.0)	16.5–16.7
Brief contacts with a GP	9.7 (8.7)	9.6–9.8	6.6 (6.7)	6.6–6.6
Consultations with a GP	7.9 (6.5)	7.8–7.9	6.2 (5.4)	6.2–6.2
Interprofessional contacts	5.6 (10.4)	5.4–5.7	3.1 (7.3)	3.0–3.1
Home visits by a GP	0.3 (1.6)	0.2–0.3	0.1 (1.1)	0.1–0.1
Administrative contacts with a GP	0.6 (1.0)	0.6–0.6	0.3 (0.8)	0.3–0.3
Other contact types with a GP	0.2 (0.8)	0.2–0.2	0.1 (0.7)	0.1–0.1

Descriptive statistics for all contact types with a GP for high-cost and non-high-cost older patients in 2019^*^ (*n* = 186,388)

GP: General Practitioner

All included patients alive by 2019–12-31. ^**^ Includes all scheduled or emergency consultations, brief contacts, home visits, interprofessional contacts between the GP and other health professions, and administrative contacts)

### Sociodemographic and clinical characteristics for high-cost and non-high-cost older patients

Table 2 presents descriptive statistics and bivariate analyses for the characteristics included in the regression model. On average, the high-cost group had substantially more GP contacts than the non-high-cost group for the included sociodemographic characteristics. For the sociodemographic characteristics, the women in both patient groups had on average two more GP contacts than men. GP contacts increased with older age groups and were highest for persons aged 85–89 years in both the high- and non-high-cost group. For both groups, participants who were single/divorced/separated/widowed had on average three more GP contacts than those who were married, had a cohabitant or partner. The number of GP contacts decreased with higher education and income, compared to those with primary school education and the lowest household income level. The high-cost group also had approximately seven more GP contacts than the non-high-cost group for the highest level of education, highest level of income and living in most central municipalities. Patients living in the least central municipalities also had more GP contacts than those living in mid- or most central municipalities. Among the clinical characteristics, the number of GP contacts increased with the presence of more main different diagnoses for both patient groups. The high-cost group with ≥5 different main diagnoses had approximately three more GP contacts than non-high-cost group in the same category. Among those with ≥5 contacts with municipal emergency medical centers, the high-cost group had on average eight more GP contacts than the non-high-cost group. Similar results were observed for the LOS. For the patients who spent four weeks or more in hospitals, the results also showed that the high-cost group had on average approximately five more GP contacts than the non-high-cost group.

**Table 2:** Descriptive statistics and bivariate analysis of characteristics and GP contacts^*^ (*n* = 186,388)

	High-cost older patients (n = 18,638)				Non-high-cost older patients (n = 167,750)			
	N (%)	Median	Mean (SD)	95% CI	N (%)	Median	Mean (SD)	95% CI
**Sex**								
Male	10,363 (56%)	19.0	23.3 (17.5)	22.9–23.6	76,723 (46%)	12.0	15.4 (12.9)	15.3–15.5
Female	8,275 (44%)	21.0	25.9 (19.7)	25.4–26.3	91,027 (54%)	14.0	17.8 (14.7)	17.7–17.9
**Age-groups**								
65–69 years	3,289 (20%)	17.0	22.2 (18.5)	21.6–22.8	32,846 (20%)	10.0	13.4 (12.2)	13.3–13.5
70–74 years	4,497 (27%)	18.0	22.6 (17.7)	22.1–23.1	39,438 (24%)	12.0	14.7 (12.6)	14.6–14.9
75–79 years	3,712 (23%)	20.0	24.0 (17.6)	23.5–24.5	32,802 (20%)	13.0	16.4 (13.2)	16.4–16.7
80–84 years	2,531 (16%)	23.0	27.3 (19.0)	26.6–28.0	26,653 (16%)	15.0	18.9 (14.6)	18.7–19.0
85–89 years	1,568 (10%)	25.0	28.7 (19.4)	27.8–29.6	20,748 (12%)	17.0	20.5 (15.7)	20.3–20.8
>90 years	629 (4%)	24.0	28.4 (21.5)	26.9–29.9	14,263 (8%)	17.0	20.4 (16.2)	20.2–20.7
**Marital status **^**1**^								
Married/Partner/Cohabitant	10,182 (55%)	19.0	22.9 (17.2)	22.6–23.2	86,356 (52%)	12.0	15.0 (12.2)	15.0–15.1
Single/Separated/Divorced/Widowed	8,456 (45%)	21.0	26.2 (19.8)	25.8–26.2	81,394(48%)	15.0	18.4 (15.4)	18.3–18.5
**Educational level **^**2**^								
Primary school	5,748 (31%)	22.0	27.1 (19.9)	26.6–27.7	49,913 (30%)	15.0	19.0 (15.4)	18.9–19.2
High school	9,203 (50%)	19.0	23.9 (18.3)	23.5–24.3	80,407 (49%)	13.0	16.4 (13.5)	16.3–16.5
Higher education (university/college)	3,509 (19%)	17.0	21.2 (16.0)	20.7–21.8	35,420 (21%)	11.0	14.0 (11.9)	14.0–14.2
**Household income**								
Low income (≤450,000 NOK)	8,121 (44%)	22.0	27.0 (20.2)	26.6–27.4	77,200 (46%)	15.0	19.0 (15.7)	18.9–19.1
Moderate income (451–750,000 NOK)	7,823 (42%)	19.0	23.2 (17.2)	22.8–23.5	65,190 (39%)	12.0	15.4 (12.2)	15.3–15.5
High income (>750,000 NOK)	2,685 (14%)	16.0	20.2 (15.6)	19.6–20.8	24,967 (15%)	10.0	12.8 (10.8)	12.7–13.0
**Municipal centrality**								
Least central municipalities	3,479 (19%)	22.0	27.0 (19.1)	26.4–27.7	29,542 (18%)	14.0	18.0 (15.0)	17.8–18.1
Mid-central municipalities	7,995 (43%)	20.0	25.2 (19.4)	24.8–25.6	72,405 (43%)	14.0	17.3 (14.4)	17.2–17.4
Most central municipalities	7,146 (38%)	18.0	22.3 (17.0)	21.9–22.7	65,308 (39%)	12.0	15.5 (12.7)	15.4–15.6
**Main diagnosis groups **^**3**^								
1 main diagnosis	1,305 (7%)	14.0	17.4 (14.5)	16.6–18.2	56,785 (34%)	10.0	12.4 (10.9)	12.3–12.4
2 different main diagnoses	3,086 (16%)	16.0	19.4 (14.5)	18.9–19.9	51,939 (31%)	13.0	16.0 (12.8)	15.9–16.2
3 different main diagnoses	4,234 (23%)	19.0	22.6 (17.3)	22.1–23.2	31,021 (19%)	16.0	19.4 (14.6)	19.2–19.5
4 different main diagnoses	3,864 (21%)	21.0	24.9 (17.5)	24.3–25.4	16,174 (10%)	18.0	22.3 (16.3)	22.1–22.6
≥5 different main diagnoses	6,149 (33%)	24.0	29.3 (21.1)	28.8–29.9	10,831 (6%)	22.0	26.0 (17.8)	25.6–26.3
Contacts with municipal emergency medical centers^*^								
No contact	6,025 (32%)	15.0	17.9 (12.8)	17.6–18.2	68,085 (40%)	11.0	13.8 (10.8)	13.7–13.9
1 contact	4,009 (22%)	17.0	21.0 (15.0)	20.6–21.5	48,028 (29%)	12.0	14.9 (12.1)	14.8–15.0
2 contacts	2,753 (15%)	21.0	24.3 (17.0)	23.6–24.9	25,503 (14%)	15.0	18.0 (14.0)	17.9–18.2
3 contacts	1,744 (9%)	23.0	27.4 (18.2)	26.5–28.2	11,268 (7%)	18.0	21.3 (15.8)	21.0–21.6
4 contacts	1,129 (6%)	26.0	30.5 (20.0)	29.4–31.7	6,365 (4%)	20.0	24.2 (17.7)	23.7–24.6
≥5 contacts	2,978 (16%)	34.0	38.3 (24.5)	37.4–29.1	10,501 (6%)	26.0	30.7 (21.4)	30.3–31.1
**Length of stay in somatic hospitals**								
≤1 week (0–6 days)	1,546 (8%)	14.0	17.1 (13.2)	16.4–17.8	134,077 (80%)	12.0	15.2 (12.7)	15.2–15.3
1–2 weeks (7–13 days)	4,450 (24%)	17.0	20.5 (15.0)	20.0–20.9	25,521 (14%)	18.0	21.4 (16.1)	21.2–21.6
2–3 weeks (14–20 days)	4,053 (22%)	20.0	24.1 (17.7)	23.6–24.7	6,279 (4%)	20.0	24.5 (18.6)	24.0–25.0
3–4 weeks (21–27 days)	2,763 (15%)	22.0	26.6 (19.5)	25.8–27.3	2,197 (1%)	21.0	25.2 (18.5)	24.5–26.0
≥4 weeks (≥28 days)	5,826 (31%)	24.0	28.6 (21.0)	28.0–29.1	1,676 (1%)	19.0	23.2 (17.0)	22.4–24.0

Descriptive statistics and bivariate analysis of characteristics and GP contacts^*^ for high-cost and non-high-cost older patients in 2019

GP: General Practitioner

^*^ Contacts include all scheduled and emergency consultations, home visits, interprofessional contacts, brief contacts, other contact types and administrative contacts

^**1**^ Divorced includes separated individuals,^**2**^ High school includes vocational school, ^3^ Includes all 22 main diagnosis groups from NPR

Bivariate analysis: Mann-Whitney U; *Comparison of the distribution of GP contacts between high-cost and non-high-cost older patients across different characteristics. The comparison groups for the bivariate analyses were the high- and non-high-cost group within each characteristic*. All significant at <0.05 except for LOS 2–3 weeks (*p* = 0.90)

### The frequency of the use of GP services

**Fig. 2:**
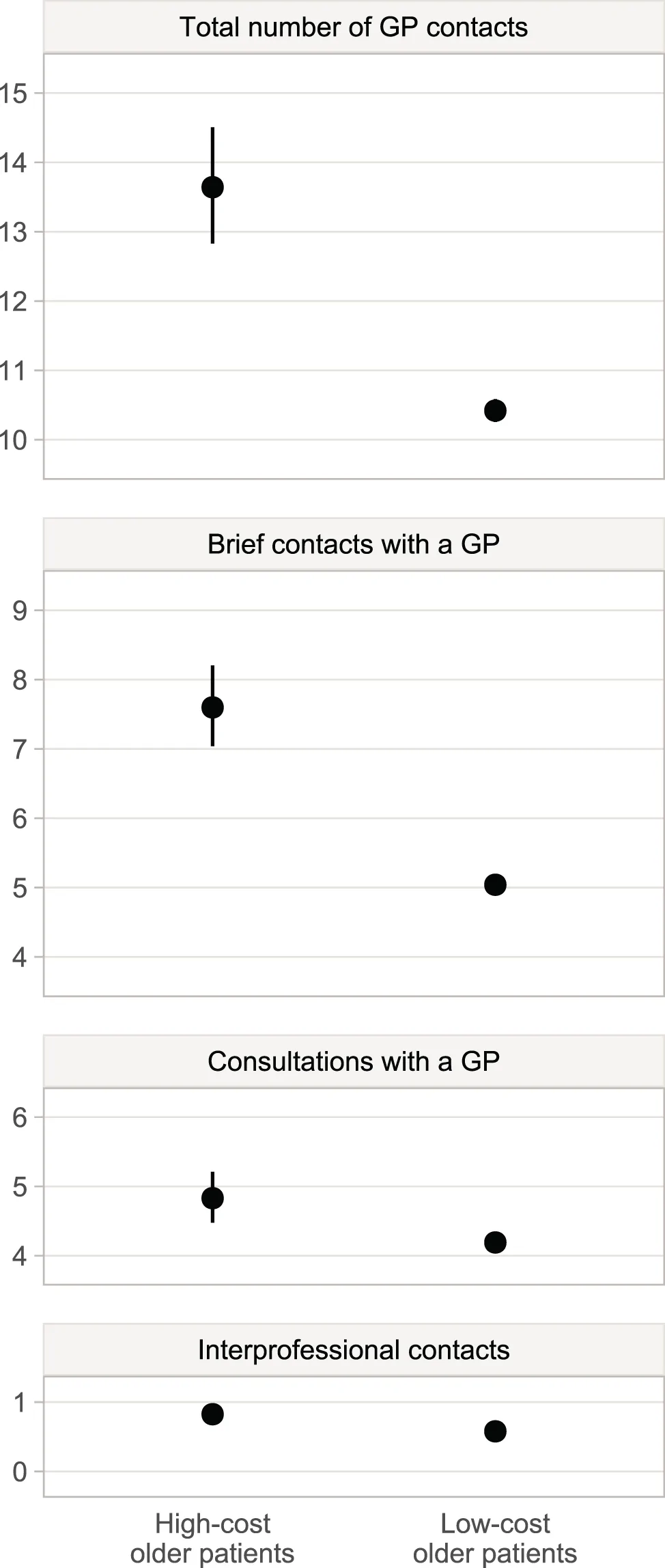
Expected number of GP contacts for a reference patient (male, 65–69 years, married/partner/cohabitant, primary school education, low income, least central municipality, 1 main diagnosis, 0 contacts with municipal emergency medical centers, <1 week in hospital) across contact types (*n* = 183,847). The results are based on a negative binomial regression model. The vertical lines represent 95% confidence intervals (for the non-high-cost older patient group, these are so narrow that they are hidden by the point estimate marker)

The results for the negative binomial regression analysis are presented in Table 3. A total of 183,847 patients were included in the analysis − 18,440 high-cost older patients and 165,407 non-high-cost older patients. The expected frequency of GP contacts for a reference patient *(male, 65–69 years, married/partner/cohabitant, primary school education, low income (≤450,000 NOK, least central municipality, 1 main diagnosis, 0 contacts with municipal emergency medical centers, <1 week in hospital (0–6 days))* was 13.64 for the high-cost group and 10.40 for the non-high-cost group. This difference (and lack of overlap in the confidence intervals) shows that even after adjusting for the different characteristics in the two groups, the high-cost group have a higher frequency of GP contacts than the non-high-cost group. Figure 2 and Appendix 3 show the expected number of GP contacts for a reference patient across various types of contacts, including total number of contacts, brief contacts, consultations with a GP and interprofessional contacts. Higher frequency of GP contacts is observed for the various contact types among the high-cost group when compared to the non-high-cost group.

**Table 3:** Regression model for the expected frequency of GP contacts^*^ among high-cost and non-high-cost older patients (*n* = 183,847)

	High-cost older patients (n = 18,440)			Non-high-cost older patients (n = 165,407)		
	exp(*β*)	95% CI	*p*-value	exp(*β*)	95% CI	*p*-value
Intercept	13.64	12.37–15.03	<0.001	10.40	10.16–10.65	<0.001
**Sex **^**a**^						
Female	1.08	1.05–1.11	<0.001	1.10	1.09–1.12	<0.001
**Age-groups **^**b**^						
70–74 years	0.99	0.95–1.04	0.71	1.05	1.03–1.06	<0.001
75–79 years	1.03	0.98–1.07	0.27	1.12	1.11–1.14	<0.001
80–84 years	1.14	1.08–1.20	<0.001	1.24	1.22–1.26	<0.001
85–89 years	1.13	1.06–1.19	<0.001	1.31	1.29–1.34	<0.001
>90 years	1.11	1.02–1.20	0.02	1.30	1.28–1.33	<0.001
**Marital status **^**c**^						
Single/Separated/Divorced/Widowed	1.02	0.98–1.06	0.37	1.04	1.03–1.06	<0.001
**Educational level **^**d**^						
High school^**^	0.94	0.91–0.98	0.001	0.93	0.92–0.94	<0.001
Higher education (university/college)	0.90	0.86–0.94	<0.001	0.86	0.85–0.88	<0.001
**Household income **^**e**^						
Moderate income (451–750,000 NOK)	0.96	0.92–0.99	0.02	0.94	0.92–0.95	<0.001
High income (>750,000 NOK)	0.89	0.85–0.94	<0.001	0.86	0.85–0.88	<0.001
**Municipal centrality **^**f**^						
Mid-central municipalities	0.95	0.91–0.99	0.01	0.98	0.97–0.99	0.01
Most central municipalities	0.85	0.81–0.89	<0.001	0.91	0.89–0.92	<0.001
**Main diagnosis groups **^**g**^						
2 different main diagnoses	1.07	1.00–1.15	0.043	1.26	1.24–1.27	<0.001
3 different main diagnoses	1.21	1.13–1.29	<0.001	1.47	1.45–1.49	<0.001
4 different main diagnoses	1.29	1.20–1.37	<0.001	1.66	1.63–1.69	<0.001
≥5 different main diagnoses	1.45	1.36–1.55	<0.001	1.89	1.85–1.94	<0.001
Contact with municipal emergency medical centers ^* h^						
1 contact	1.12	1.07–1.17	<0.001	1.04	1.03–1.05	<0.001
2 contacts	1.24	1.19–1.30	<0.001	1.19	1.17–1.20	<0.001
3 contacts	1.36	1.28–1.43	<0.001	1.33	1.30–1.36	<0.001
4 contacts	1.50	1.40–1.60	<0.001	1.46	1.42–1.50	<0.001
≥5 contacts	1.80	1.71–1.88	<0.001	1.78	1.75–1.82	<0.001
**Length of stay in somatic hospitals **^**i**^						
1–2 weeks (7–13 days)	1.12	1.05–1.19	<0.001	1.16	1.15–1.18	<0.001
2–3 weeks (14–20 days)	1.20	1.13–1.28	<0.001	1.24	1.21–1.27	<0.001
3–4 weeks (21–27 days)	1.26	1.18–1.35	<0.001	1.25	1.20–1.31	<0.001
≥4 weeks (≥28 days)	1.31	1.23–1.39	<0.001	1.17	1.11–1.23	<0.001

Negative binomial regression analysis of GP contacts by characteristics, stratified by high-cost and non-high-cost older patients in 2019

GP: General Practitioner

2,541 participants (1,4%) with missing information on educational level, household income or index of municipal centrality were automatically excluded from the regression analysis

^*^ Contacts include all scheduled and emergency consultations, home visits, interprofessional contacts, brief contacts, other contact types and administrative contacts

^**^ Educational level (high school includes vocational school)

Reference level: ^a^ (male), ^b^ (65–69 years), ^c^ (married, registered partner, cohabitant), ^d^ (primary school), ^e^ (Low income: ≤ 450,000 NOK), ^f^ (least central municipalities), ^g^ (1 main diagnosis), ^h^ (no contacts with municipal emergency medical centers), ^i^ (< 1 week 0–6 days)

Several predictors were associated with higher expected frequency of GP contacts, including being female, being in the oldest age groups, having multiple different main diagnoses, having multiple contacts with a municipal emergency medical center, and having longer LOS in somatic hospitals. For the high-cost group, being female was associated with having 8% more expected GP contacts than being male (exp(*β*) 1.08, *p* < 0.001). Patients aged 80–85 years, 85–89 years, and 90 years and older had 14, 13, and 11% more expected GP contacts, respectively, than patients aged 65–69 years. Compared to those having a primary school education, having a higher education was associated with 10% fewer GP contacts. Having the highest income level was associated with 11% fewer GP contacts among the high-cost group. We saw similar results for those with the highest income levels compared to those with the lowest income level. For those residing in the most central municipalities, the high-cost group had 15% fewer expected GP contacts than those residing in the least central municipalities. In the high-cost group, those with ≥different 5 main diagnoses had 45% more expected GP contacts than patients with only one main diagnosis. Having multiple out-of-hours contacts with a municipal emergency medical center was associated with a substantially higher GP contact frequency. For example, the high-cost group with ≥5 contacts with a municipal emergency medical center had 80% more expected GP contacts than those with no contacts with a municipal emergency medical center. We also found that longer stays in somatic hospitals were associated with a higher expected frequency of GP contacts, where the high-cost group admitted ≥ four weeks had 31% more expected GP contacts than those with an LOS ≤ one week

Similarly, for the non-high-cost group, we found that women had 10% more expected GP contacts than men. Patients aged 85–89 and 90 years and older had respectively 31 and 30% more expected GP contacts than those aged 65–69 years. Having five or more different main diagnoses was associated with 89% higher expected GP contact frequency than having one main diagnosis. In the non-high-cost group, those with ≥5 contacts with a municipal emergency medical center had 78% more expected GP contacts than those with no municipal emergency medical center contacts. Patients admitted for four weeks or longer in somatic hospitals had 17% more expected GP contacts than those admitted for <1 week. Compared to those having a primary school education, having a higher education was associated with 14% fewer GP contacts for the non-high-cost group. Similar results were observed for income levels, where the non-high-cost group with the highest income level had 14% fewer expected GP contacts compared to those with the lowest income level. For those residing in the most central municipalities, the non-high-cost group had 9% fewer contacts than those residing in the least central municipalities.

### Sensitivity and supplemental analyses

When excluding participants with a high number of GP contacts (≥50) in the sensitivity analysis, we observed similar results for the regression analysis, with only minor variations in the regression coefficients. This was also the case when including participants who died during 2019 in the regression model and running the analysis without the income variable. The supplemental analyses showed similar associations as in the main analysis across all variables when examining the contact subgroups: consultations, brief contacts and interprofessional contacts (results not shown). The descriptive and bivariate analysis for the deceased participants is presented in Appendix 4.

## Discussion

This study aimed to explore the frequency of the use of GP services among high-cost older patients and other older patients admitted to Norwegian somatic hospitals and to identify characteristics associated with the frequency of service use. We found that the high-cost group utilized significantly more GP services than the non-high-cost group, including brief contacts, consultations, and interprofessional contacts. The average number of GP consultations in our study sample for both the high- and non-high-cost group exceeded the national average for patients aged 65 years and older in 2019 [24]. This may be due to all participants having at least one unplanned contact with somatic hospitals which might require some form of GP follow-up. While the differences in GP consultations between the groups are relatively modest, the disparities are more pronounced among other contact types, such as brief contacts and interprofessional contacts. The differences in brief contacts and interprofessional contacts may reflect the complex and frequent need for coordination and follow-up care among high-cost older patients [39]. As the high-cost group overall have substantially more GP contacts than the non-high-cost group, we argue that they should be deemed *high utilizers* or *frequent users of GP services*. Similar findings are reported by Dreyer et al. [40], who found that high-cost, high-need patients tend to use both GP and hospital services more frequently than other patients, driven by their multiple and complex medical conditions. It is important to note that hospital service utilization may inherently overlap with GP service use. The gatekeeping and coordinating role of GPs might involve patient contact both before and after hospital admission [5]. However, our findings illustrate that the high healthcare utilization associated with high-cost older patients extends beyond the context of somatic hospitals, aligning with findings from previous studies [9, 15].

High use of GP services does not guarantee that their healthcare needs are adequately met, as high utilization does not necessarily imply high quality of care. Nonetheless, the higher number of interprofessional contacts among the high-cost group in our study might indicate that they require more integrated care than other older adult patients [41]. Hansen et al. [7] found that continuity of GP care was associated with reduced use of specialist services, suggesting that GPs have a major coordinating role in managing healthcare needs before hospital care becomes necessary. In contrast, other studies have reported that high use of hospital resources and frequent contact with GPs could reflect that GPs have a higher referral rate for those with complex conditions [42, 43]. While we are unable to examine this in our study, we argue that the complexity of high-cost older patients’ health conditions often necessitates specialized care, regardless of GP follow-up, as highlighted in previous research [11, 44].

Regarding the characteristics associated with the frequency of GP service use, we found some variations that should be discussed. The observed associations between sex, older age, and higher use of GP services in our study are consistent with results from previous studies [45–49]. According to previous research, there is a notable difference in how men and women use healthcare services. This variation often stems from differences in healthcare needs and healthcare-seeking behavior [45, 47], as women tend to have higher use of primary healthcare, including GP services, while men have higher use of specialized healthcare services [50, 51]. A recent Norwegian study also revealed that the burden of disease and overall healthcare utilization are significantly higher among older women (≥65 years) than among older men [49]. This finding also corresponds with the results of a previous study, which indicated that men used more somatic hospital services and were more likely to be classified as high-cost older patients, than women were [26]. GP service utilization often increases with age due to frailty, physical decline, and more chronic conditions that often require frequent GP follow-up [41]. Both the high-cost and non-high-cost groups show increased use of GP services with advancing age with the high-cost group having a higher number of GP contacts across all age categories. The ratio of the expected number of GP contacts in the older age groups versus the youngest age groups is larger for the non-high-cost older patients. One reason might be that they start from a lower baseline. This steeper relative increase may reflect a convergence in healthcare needs between the two groups as they age.

Regarding the socioeconomic factors associated with the frequency of GP service use, we found that higher education and high household income were associated with less frequent use of GP services. This result is consistent with Norwegian and international studies showing that older adults with low income and lower education tend to use more GP services than those with higher education and income [52, 53]. A public health report has shown that inequalities in health are apparent across income and education levels in Norway [54]. Those with low income and education often have poorer health outcomes and greater healthcare needs than those with high education and income. However, the high use of GP services among those with low socioeconomic status might also indicate that the Norwegian welfare system is functioning as intended. While there are disparities in health outcomes, everyone should have equal access to healthcare services regardless of their capacity to pay [20]. Given the results, this might indicate that educational and financial constraints do not limit access to GP services for either high-cost or non-high-cost older patients. It is important to note that individuals with higher socioeconomic status may not necessarily have lower utilization of healthcare services but rather opt for different types of services. For instance, they might have better access to private healthcare providers, bypassing the gatekeeping role of GPs for less severe conditions that could be treated in public specialized healthcare services. This could reflect a divergence in the type of healthcare services utilized rather than an overall lower need for healthcare, as seen in previous Nordic studies [55, 56]. We also observed that older patients living in less central municipalities had higher use of GP services than those living in more central municipalities. Many of the least central municipalities in Norway are located remotely from public entities such as hospitals. Less central municipalities often have smaller populations [36, 57]. In these municipalities, the GP coverage per resident is higher [58]. This, in combination with long distances to specialized services, might contribute to higher use of GP services in these municipalities [2].

In this study, a higher number of different main diagnoses and contacts with a municipal emergency medical center were associated with higher use of GP services for both patient groups. According to previous research, high-cost older patients often have complex healthcare needs that are associated with both high use of specialized care and municipal emergency medical center services [9, 12, 44, 59]. In addition, high-cost older patients often have conditions affecting the circulatory and respiratory system, such as coronary heart disease, heart failure and chronic obstructive pulmonary disease [60]. These conditions may cause exacerbations requiring acute care, whereas other older adults may have less severe diagnoses that are more manageable through GP services during regular hours. A prior study has found a higher prevalence of different main diagnoses affecting the circulatory, respiratory, neurological, and gastrointestinal organs among high-cost older patients than among other older adults in somatic hospitals [26]. This is also consistent with several other studies, showing that multiple diagnoses within these main diagnosis groups are among the most common causes of municipal emergency medical center visits [5, 45, 61]. Overall, these studies illustrate that the healthcare needs of patients with acute and complex medical conditions may be challenging to manage during regular hours of general practice.

Given the heterogeneous and complex nature of high-cost older patients’ treatment, it may not be possible to adapt a general strategy for reducing the healthcare utilization among these patients. Instead, the objective should be to provide equitable access to services and improved integrated care for these patients to prevent negative health outcomes and reduce the risk of unnecessary hospitalizations. This inherently means that service use will vary, ensuring that those with the greatest needs receive the most care. While several strategies exist, researchers have argued that healthcare for high-cost older patients should primarily involve a person-centered, interprofessional and holistic approach in all aspects of healthcare delivery [62, 63]. While our data are not suited for analyzing the collaboration between healthcare providers, a previous study indicate that there still are challenges in this area [64]. Thus, future studies should investigate the management and effectiveness of collaboration. Analyzing the implementation of integrated care models could provide valuable insights. By comparing health outcomes and healthcare utilization between high-cost older patients in integrated care settings and in traditional care settings, the suitability of such models in managing complex patient needs may be assessed. Additionally, mapping the healthcare pathways for high-cost older patients can provide insights helpful for adapting patient management, ensuring timely and appropriate interventions.

### Strengths, limitations, and implications for future research

The main strength of this study is that the data are derived from national registers that cover all older adults with at least one unplanned contact with somatic hospitals during an entire year. Due to the large sample sizes, we get precise estimates of all quantities and associations. The data provide comprehensive information on an individual level which generates important knowledge about variations in GP service use. Using a negative binomial regression to examine the expected frequency of GP contacts is a strength to the validity of the results as the model accounts for overdispersion in the data and allows us to consider differences in individual patient characteristics. It is a strength that the high-cost group is defined using DRG weights, as this allows for comparisons with other healthcare systems using DRG measurements. Classifying patients in two groups as either high-cost or non-high-cost provides a clear comparison between the groups, but this approach also has limitations. Our data is not suited to differentiate between healthcare needs and utilization of services. The results should thus be interpreted with caution, especially when drawing conclusions about the healthcare needs of high-cost older patients. By focusing on the top 10%, we may not capture variations in healthcare cost just below the 10% cut-off, potentially overlooking individuals with significant healthcare utilization. The findings might not apply to other settings with different criteria for defining high-cost populations, as our analysis is limited to older adults with unplanned hospital contacts. Using broader inclusion criteria could yield different results. Examining the entire older adult population with both planned and unplanned hospital visits could provide further insights into healthcare utilization.

Given the cross-sectional design of our study, we were unable to examine any causal relationships between patient characteristics and the frequency of GP service use. The single-year timeframe prevents us from examining the predictors of service utilization longitudinally. Additionally, we are unable to examine whether the high use of GP services among the high-cost group is a direct consequence of their substantial resource use in hospitals. To investigate this issue, more detailed data on the timing of hospital admissions and GP visits are needed. The exclusion of patients who died during 2019 may introduce selection bias, which theoretically could affect the generalizability of our findings. As we performed sensitivity analyses, we considered this risk to be low. Furthermore, while we included several variables reflecting sociodemographic and clinical characteristics in our analysis, other potential influencing variables such as ethnicity, drug use, lifestyle, and cultural background were not available. The inclusion of such variables could have presented a more detailed picture of the overall health status of the participants and associations with GP service use. As our data limit us from examining the quality or effectiveness of GP services in meeting the needs of high-cost older patients, this topic should be the subject of future research. Future studies should examine how various medical and care needs could affect healthcare utilization among these patients and should consider using longitudinal studies that evaluate the impact of integrated care models, quality of care and structured follow-up programs. In this context, studies should also examine how these patients utilize other municipal healthcare services such as homebased care and/or institutional services, such as nursing homes. Such research would allow for a more comprehensive assessment of how healthcare outcomes can be improved for high-cost older patients and provide more definitive conclusions about effective strategies for healthcare optimization.

## Conclusion

The study indicates that high-cost older patients had significantly higher utilization of GP services compared to other older adults admitted to Norwegian somatic hospitals in 2019. High-cost older patients often have multimorbidity and complex health issues that may require more frequent GP attention, and they could be deemed frequent users of GP services. We further found that older age, female sex, multiple diagnoses, multiple contacts with a municipal emergency medical center, and long hospital stays were associated with higher use of GP services, which might indicate that groups known to have higher healthcare needs might also use more services. This highlights the importance of equitable access to healthcare, which is an essential goal in the universal healthcare system.

## Appendix 1: Description of contact type with GP or municipal emergency medical clinics with tariff/rate-codes

**Table:** 

Contact type	Description	Tariff code
Consultation (scheduled and emergency)	Contacts where the patient has a consultation with a GP, either face-to-face or digitally	
	• Consultations with a GP • Group therapy • Interdisciplinary consultations via video and/or telephone between GPs and healthcare personnel in the municipal health and care services, and/or ambulance personnel	Consultations with a physician: 2ad, 2ak, 2ed, 2fk, 615 E-consultations with a physician: 2ae
Brief contacts	Contacts that are less comprehensive than consultations	
	• Brief contacts with a patient (daytime or evening) • Brief contacts with a patient; electronically, by letter or phone • Sick note / sick leave and referral without a patient present • Writing of electronic prescriptions (e-prescriptions) • Collection of lab samples at GP’s office or submission to medical laboratory • Necessary conversation with relatives of a patient with a serious illness or intellectual disability, or with guardians of children under 18 years of age • Supervised urine sample collection for patients in LAR (medication-assisted treatment for substance dependency) • Vaccination against seasonal influenza for individuals in risk groups	Brief contacts with the patient present: 1ad, 1ak, 1e, 618,701a Brief contacts through letter, phone or administration: 1bd, 1bk, 1 g, 1 h, 1i, 612a, 612b Brief electronic contacts: 1be
Home visits	Contacts where the GP visits the patient in their home	
	• Home visits (daytime or evening)	Home visits by a physician: 11ad, 11ak, 11bd, 11 cd, 12ad, 12bd, 12 cd, 13d
Interprofessional contacts	Contacts where the GP and an interprofessional group address the needs of a patient with complex conditions	
	• Communication with healthcare professionals / municipal healthcare services / NAV (Norwegian Labor and Welfare Administration) • Interdisciplinary collaboration meeting • Necessary dialogue between GPs and specialist healthcare providers	Brief contact with other healthcare professions/municipal services/NAV^*^: 1f Interprofessional collaboration meetings: 14 Additional tariffs/rates interprofessional collaboration meetings: 14d, 15a, 15b Dialogue meetings NAV^*^: L35, L35d, L36d Necessary dialogue between GPs and specialized healthcare: 1j
Administrative contacts/other contacts	Contacts involving administrative tasks such as writing certificates or other documents and other tasks that do not fall under the groupings above	
	• Writing information for the national summary care record (*kjernejournal*) • Particularly time-consuming work related to referrals or admissions • Printing or copying patient records	Selected tariffs for administrative work: H1, 5, 7, 8, 2kd, 2ld, L-tariffs

Definition of contact type with GP or municipal emergency medical centers with tariff/rate-code for reporting to the Norwegian Health Economics Administration (HELFO)

NAV: Norwegian Labour and Welfare Administration

This information is collected from the Norwegian Directorate of Health’s report on the use of general practitioner and municipal emergency medical services 2017-2019. Statistics and Analysis of Health Services in Municipalities (SAMDATA). Available from: https://www.helsedirektoratet.no/rapporter/bruk-av-fastlege-og-legevakt-2017-2019/

## Appendix 2: Main Diagnosis Groups from the Norwegian Patient Registry

**Table:** 

Information about the variable(s)
The number of times this main diagnosis group in the DRG-system was registered with each patient in 2019
HDG: Hoved diagnose gruppe (Norwegian): Main Diagnosis Group (English)
NPR: Norwegian Patient Registry
NPR – HDG – Diseases of the nervous system
NPR – HDG – Diseases in breast tissue
NPR – HDG – Diseases of the female genitalia
NPR – HDG – Ear, nose, and throat disorders
NPR – HDG – Diseases of the respiratory organs
NPR – HDG – Diseases of the circulatory organs
NPR – HDG – Diseases of the blood, blood-forming organs, and immune system
NPR – HDG – Diseases of the digestive organs
NPR – HDG – Myeloproliferative disorders and differentiated tumors
NPR – HDG – Categories across multiple diagnostic groups
NPR – HDG – Infections and parasitic diseases
NPR – HDG – Burns and burn related wounds
NPR – HDG – Categories for error and uncommon diagnosis procedure combinations
NPR – HDG – Diseases of the liver, biliary tract, and pancreas
NPR – HDG – Mental disorders and substance abuse problems
NPR – HDG – Diseases of the musculoskeletal system and connective tissue
NPR – HDG – Trauma, poisoning and toxic effect of drugs/other substances, drug abuse and organic mental disorders
NPR – HDG – Diseases of the skin and subcutaneous tissue
NPR – HDG – Internal secretory, nutritional, and metabolic disorders
NPR – HDG – Kidney and urinary tract disorders
NPR – HDG – Diseases of the male genitalia

## Appendix 3: Adjusted regression analysis results for various GP contact types for a reference patient

**Table:** 

	High-cost older patients (n = 18,440)		Non-high-cost older patients (n = 165,407)	
	exp(*β*)	95% CI	exp(*β*)	95% CI
**GP all contacts ** ^**1**^				
Intercept	13.64	12.37–15.03	10.40	10.16–10.65
**GP brief contacts**				
Intercept	7.59	6.87–8.39	5.03	4.91–5.16
**GP consultations ** ^**2**^				
Intercept	4.83	4.36–5.35	4.19	4.08–4.29
**GP interprofessional contacts**				
Intercept	0.83	0.74–0.94	0.64	0.62–0.66

Regression analysis results adjusted for sex, age-groups, marital status, educational level, household income, number of main diagnosis groups, number of contacts with municipal emergency medical centers and LOS, for various GP contact types for the reference patient ^*^ (*n* = 183,847)

_*_ Reference level: ^a^ (male), ^b^ (65–69 years), ^c^ (married, registered partner, cohabitant), ^d^ (primary school), ^e^ (Low income: ≤450,000 NOK), ^f^ (least central municipalities), ^g^ (1 main diagnosis), ^h^ (no contacts with municipal emergency medical centers), ^i^ (<1 week 0–6 days)

GP: General Practitioner

2,541 participants (1,4%) with missing information on educational level, household income or index of municipal centrality were automatically excluded from the regression analysis

^**1**^ All contacts include all scheduled and emergency consultations, home visits, interprofessional contacts, brief contacts, other contact types and administrative contacts

^**2**^ Consultations include all scheduled and emergency consultations

## Appendix 4: Descriptive and bivariate analysis of characteristics and GP contacts* for deceased participants

**Table:** 

	High-cost older patients (n = 5,381)				Non-high-cost older patients (n = 15,550)			
	N (%)	Median	Mean (SD)	95% CI	N (%)	Median	Mean (SD)	95% CI
**Sex**								
Male	3,072 (57%)	14.0	18.7 (16.6)	18.1 – 19.3	7,607 (49%)	8.0	12.1 (13.4)	11.8 – 12.4
Female	2,309 (43%)	14.0	18.8 (17.0)	18.1 – 19.5	7,943 (51%)	8.0	12.4 (13.4)	12.2 – 12.7
**Age-groups**								
65-69 years	877 (16%)	12.0	16.5 (15.3)	15.5 – 17.6	912 (6%)	8.0	11.8 (12.9)	10.9 – 12.6
70-74 years	1,256 (24%)	12.0	17.3 (16.7)	16.4 – 18.3	1,774 (11%)	8.0	11.9 (13.4)	11.3 – 12.5
75-79 years	1,187 (22%)	14.0	18.1 (15.7)	17.2 – 19.0	2,138 (14%)	8.0	11.6 (12.4)	11.1 – 12.2
80-84 years	982 (18%)	15.0	19.9 (17.5)	18.8 – 21.0	2,789 (18%)	8.0	12.4 (13.4)	11.9 – 12.9
85-89 years	663 (12%)	17.0	22.0 (17.5)	20.6 – 23.3	3,613 (23%)	9.0	12.9 (13.9)	12.5 – 13.4
> 90 years	416 (8%)	17.5	21.6 (18.3)	19.8 – 23.4	4,324 (28%)	8.0	12.3 (13.6)	11.9 – 12.7
**Marital status ** ^**1**^								
Married/Partner/Cohabitant	2,860 (53%)	14.0	18.6 (16.8)	18.0 – 19.2	5,890 (38%)	8.0	12.3 (13.2)	11.9 – 12.6
Unmarried/Divorced/Widowed	2,521 (47%)	14.0	18.9 (16.6)	18.3 – 19.6	9,660 (62%)	8.0	12.4 (13.6)	12.1 – 12.6
**Educational level ** ^**2**^								
Primary school	1,754 (33%)	15.0	19.7 (17.3)	18.9 – 20.5	6,256 (41%)	8.0	12.6 (13.7)	12.3 – 12.9
High school	2,655 (50%)	14.0	18.6 (16.4)	18.0 – 19.2	6,989 (46%)	8.0	12.2 (13.2)	11.9 – 12.5
Higher education (university/college)	927 (17%)	13.0	17.5 (16.5)	16.4 – 18.6	2,103 (13%)	8.0	11.8 (13.5)	11.2 – 12.4
**Household income**								
Low income (≤ 450,000 NOK)	…	…	…	…	…	…	…	…
Moderate income (451-750,000 NOK)	…	…	…	…	…	…	…	…
High income (>750,000 NOK)	…	…	…	…	…	…	…	…
**Municipal centrality**								
Least central municipalities	911(17%)	18.0	21.8 (17.7)	20.7 – 23.0	2,924 (19%)	9.0	13.6 (13.9)	13.0 – 14.0
Mid-central municipalities	2,282 (42%)	16.0	20.2 (17.3)	19.5 – 20.9	6,959 (45%)	9.0	13.0 (14.1)	12.7 – 13.4
Most central municipalities	2,184 (41%)	12.0	16.0 (15.2)	15.3 – 16.6	5,647 (36%)	7.0	10.8 (12.2)	10.5 – 11.1
**Main diagnosis groups ** ^**3**^								
1 main diagnosis	554 (10%)	7.0	10.6 (11.6)	9.6 – 11.5	6,693 (43%)	5.0	8.34 (10.3)	8.1 – 8.6
2 different main diagnoses	1,028 (19%)	10.5	14.9 (14.4)	14.0 – 15.7	4,673 (30%)	9.0	12.7 (13.3)	12.3 – 13.0
3 different main diagnoses	1,232 (23%)	14.0	18.2 (16.2)	17.3 – 19.1	2,534 (16%)	13.0	16.9 (15.0)	16.3 – 17.5
4 different main diagnoses	1,096 (21%)	16.0	20.1 (16.4)	19.1 – 21.1	1,037 (7%)	16.0	19.2 (15.4)	18.2 – 20.1
≥ 5 different main diagnoses	1,471 (27%)	19.0	23.9 (18.7)	23.0 – 24.9	613 (4%)	19.0	21.9 (17.2)	20.6 – 23.3
**Contacts with municipal emergency medical center** ^*^								
No contact	1,367 (26%)	9.0	12.1 (11.3)	11.5 – 12.7	4,055 (26%)	7.0	9.8 (9.7)	9.5 – 10.0
1 contact	1,152 (21%)	11.0	15.1 (14.1)	14.3 – 15.9	4,187 (27%)	6.0	9.7 (11.7)	9.3 – 10.0
2 contacts	829 (15%)	14.0	18.6 (15.9)	17.5 – 19.7	2,670 (17%)	8.0	11.7 (12.2)	11.2 – 12.2
3 contacts	597 (11%)	18.0	20.3 (15.0)	19.1 – 21.5	1,667 (11%)	11.0	14.1 (14.2)	13.4 – 14.8
4 contacts	422 (8%)	19.0	22.5 (16.3)	21.0 – 24.0	1,026 (7%)	12.0	15.8 (14.9)	14.9 – 16.7
≥ 5 contacts	1,014 (19%)	24.0	29.4 (21.2)	28.1 – 30.7	1,945 (12%)	17.0	20.7 (18.7)	19.8 – 21.5
**Length of stay in somatic hospitals**								
≤ 1 week (0-6 days)	306 (6%)	7.0	10.5 (10.8)	9.3 – 11.8	7,727 (49%)	6.0	10.4 (12.6)	10.1 – 10.6
1 week (7-13 days)	752 (14%)	11.0	15.4 (14.8)	14.3 – 16.4	4,260 (27%)	10.0	13.8 (13.9)	13.4 – 14.2
2 weeks (14-20 days)	1,042 (19%)	15.0	19.1 (17.1)	18.0 – 20.1	1,975 (13%)	11.0	14.6 (13.6)	14.0 – 15.2
3 weeks (21-27 days)	958 (18%)	15.0	19.7 (16.9)	18.6 – 20.8	869 (6%)	11.0	15.1 (13.5)	14.2 – 16.0
≥ 4 weeks (≥ 28 days)	2,323 (43%)	16.0	20.4 (17.3)	19.7 – 21.1	719 (5%)	10.0	14.4 (15.0)	13.3 – 15.5

Descriptive statistics and bivariate analysis of characteristics and GP contacts^*^ for deceased high-cost and non-high-cost older patients in 2019 (n = 20,931)

^*^ Contacts include all consultations (scheduled and emergency), home visits, interprofessional contacts, brief contacts, other contact types and administrative contacts

^1^ Divorced includes separated individuals, ^2^ High school includes vocational school. ^3^ Includes all 22 main diagnosis groups from NPR

Bivariate analysis: Mann-Whitney U; Comparison of the distribution of GP contacts between high-cost and non-high-cost older patients across different characteristics. The comparison groups for the bivariate analyses were the high- and non-high-cost group within each characteristic. All significant at < 0.05 except 4 MDGS (p = 0.30)

No valid measure for Household income as Statistics Norway does not collect income information for the year a person dies

Missing values (n = 270): Educational level (High-cost older patients 45, Non-high-cost older patients 202), Municipal centrality (High-cost patients 4, Non-high-cost patients 19)

## Data Availability

The dataset used in the current study is not publicly available due to the contractual arrangement with the Norwegian Directorate of Health. Researchers interested in accessing the dataset may request access through the appropriate channels established by the Norwegian Institute of Public Health which is currently the custodian of the data from January 1^st^, 2024.

## Declarations

### Authors’ information

Data from the Norwegian Patient Registry and Norwegian Registry for Primary Health Care has been used in this publication. The interpretation and reporting of these data are the sole responsibility of the authors, and no endorsement by the Norwegian Patient Registry or the Norwegian Registry for Primary Health Care is intended nor should be inferred.

## Abbreviations

GP: General practitioner
DRG: Diagnosis-related groups
LOS: Length of stay
NPR: Norwegian Patient Registry
NRPHC: Norwegian Registry for Primary Health Care
KUHR: Norwegian Control and Payment of Health Reimbursements Database
SSB: Statistics Norway
exp(*β*): Exponentiated coefficient
CI: Confidence Interval
